# P-2163. Inconsistent identification of *Corynebacterium* species by MALDI-TOF MS and molecular characteristics of *Corynebacterium jeikeium* complex isolates causing invasive infections in Japan

**DOI:** 10.1093/ofid/ofae631.2317

**Published:** 2025-01-29

**Authors:** Sohei Harada, Kohji Komori, Kenya Yukawa, Brian Hayama, Kageto Yamada, Asako Doi, Tomoo Saga, Masakazu Sasaki, Yoshiro Hadano, Yohei Doi, Yohei Doi, Kyoko Yokota, Jun Suzuki, Koki Kikuchi, Kazuhiro Tateda

**Affiliations:** Toho University School of Medicine, Ota-ku, Tokyo, Japan; Toho University School of Medicine, Ota-ku, Tokyo, Japan; Cancer Institute Hospital, Japanese Foundation for Cancer Research, Koto-ku, Tokyo, Japan; Cancer Institute Hospital, Japanese Foundation for Cancer Research, Koto-ku, Tokyo, Japan; Toho University School of Medicine, Ota-ku, Tokyo, Japan; Kobe City Medical Center General Hospital, Kobe, Hyogo, Japan; Akita University Hospital, Akita, Akita, Japan; Toho University Omori Medical Center, Ota-ku, Tokyo, Japan; Shimane University Hospital, Izumo, Shimane, Japan; Fujita Health University, Aichi, Aichi, Japan; Fujita Health University, Aichi, Aichi, Japan; Kagawa University Hospital, Kita-gun, Kagawa, Japan; Gifu Prefectural General Medical Center, Gifu, Gifu, Japan; Teine Keijinkai Hospital, Sapporo, Hokkaido, Japan; Toho University, Tokyo, Not Applicable, Japan

## Abstract

**Background:**

*Corynebacterium* spp. is increasingly identified due to widespread use of MALDI-TOF MS in clinical laboratories. However, accuracy of identification of *Corynebacterium* spp. and genetic characteristics of clinical isolates are unclear.

**Figure:**
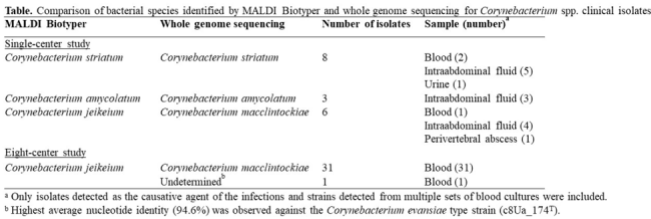

**Methods:**

We collected 17 isolates from cases diagnosed as infections caused by *Corynebacterium* spp. at a single center in Japan. Species identification was performed using MALDI Biotyper with reference library BDAL v11 (Bruker). Whole-genome sequencing (WGS) was also performed using MiSeq (Illumina), and the species were genetically identified using a threshold of ≥ 95% average nucleotide identity (ANI) with type strains. Regarding *Corynebacterium jeikeium* complex, an additional analysis was performed using 32 isolates detected from multiple sets of blood cultures at eight additional centers in Japan.

**Figure.**
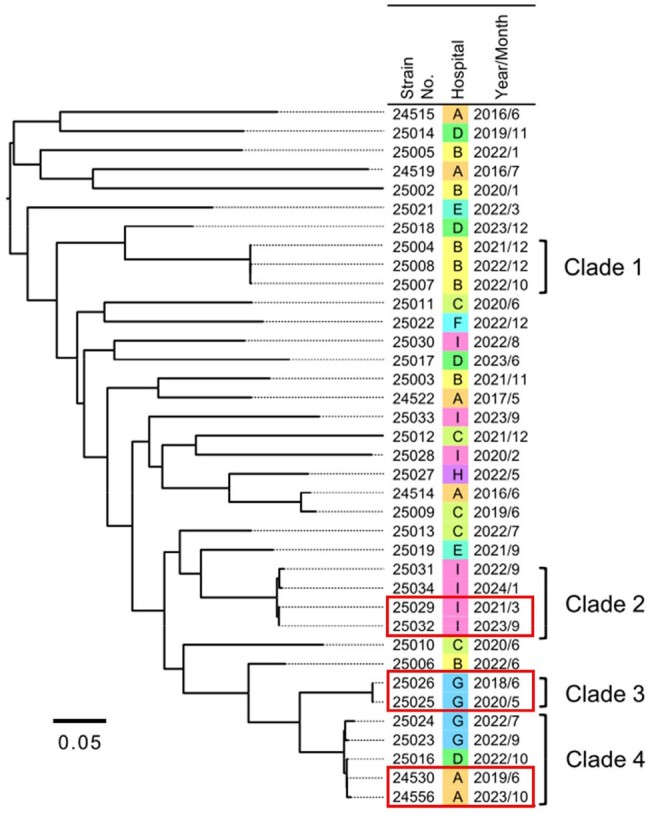
Phylogenetic tree of Corynebacterium macclintockiae clinical isolates collected in this study. Pairs of strains detected at the same facility with SNP differences of < 20/genome per year difference in isolation time are marked with red squares.

**Results:**

In the single-center study, eight isolates (2: blood, 5: intraabdominal fluid, 1: urine) and three isolates (3: intraabdominal fluid) were identified as *Corynebacterium striatum* and *Corynebacterium amycolatum*, respectively, by MALDI Biotyper, and the identification was confirmed by WGS. However, all six isolates identified as *C. jeikeium* by MALDI Biotyper (1: blood, 4: intraabdominal fluid, 1 perivertebral abscess) had an ANI of 87-88% with *C. jeikeium* NCTC 11915^T^ and were genetically identified as *Corynebacterium macclintockiae* with ANI of 97-98% with *C. macclintockiae* c9Ua_112^T^ (Table). Of the 32 isolates detected in blood cultures at eight institutions and identified as *C. jeikeium* by MALDI Biotyper, 31 were identified as *C. macclintockiae* by WGS (Table). Core-genome phylogenetic analysis of 37 *C. macclintockiae* isolates identified multiple clades spread in the same center and three pairs of isolates with SNP differences of < 20/genome per year difference in isolation time (Figure).

**Conclusion:**

Identification of *C. striatum* and *C. amycolatum* by MALDI Biotyper was consistent with the genetic identification. The vast majority of *C. jeikeium* complex isolates causing infections in Japan were genetically identified as *C. macclintockiae*, but MALDI Biotyper identified them as *C. jeikeium* with high score values. SNP analysis suggested that some *C. macclintockiae* isolates may spread by nosocomial transmission.

**Disclosures:**

Sohei Harada, MD, PhD, MSD: Honoraria|Shionogi: Honoraria Tomoo Saga, M.D., Ph.D., Asahi Kasei Pharma: Honoraria|Beckman-Coulter: Honoraria|Miyarisan Pharmaceutical: Honoraria|Pfizer: Honoraria|Shionogi: Honoraria|Sumitomo Pharma: Honoraria Yohei Doi, MD, PhD, bioMerieux: Lecture fees|Entasis: Grant/Research Support|Fujifilm: Advisor/Consultant|Gilead Sciences: Advisor/Consultant|GSK: Advisor/Consultant|KANTO CHEMICAL CO.,INC.: Grant/Research Support|KANTO CHEMICAL CO.,INC.: Patent for genotyping kit|MeijiSeika Pharma: Advisor/Consultant|Moderna: Advisor/Consultant|MSD: Lecture fees|Pfizer: Advisor/Consultant|Shionogi & Co., Ltd.: Grant/Research Support|Shionogi & Co., Ltd.: Lecture fees Kyoko Yokota, M.D, MSc, Chugai pharmaceutical Co.,Ltd: Honoraria|Meiji Seika Pharma Co.,Ltd: Honoraria Jun Suzuki, M.D., Eiken Chemical: Honoraria|Kyorin Pharmaceutical: Honoraria Koki Kikuchi, M.D., bioMérieux: Honoraria|Pfizer: Honoraria Kazuhiro Tateda, PHD, GSK: Honoraria|Moderna: Honoraria|Pfizer: Honoraria|Shionogi: Honoraria

